# Clinical Evaluation of a New Antigen-Based COVID-19 Rapid Diagnostic Test from Symptomatic Patients

**DOI:** 10.3390/diagnostics11122300

**Published:** 2021-12-08

**Authors:** Saiful Arefeen Sazed, Mohammad Golam Kibria, Mohammad Sharif Hossain, Md Fahad Zamil, Pranob Chandra Adhikary, Mohammad Enayet Hossain, Dilruba Ahmed, Rashidul Haque, Mohammad Shafiul Alam

**Affiliations:** International Centre for Diarrhoeal Disease Research Bangladesh (icddr,b), 68 Shaheed Tajuddin Ahmed Sarani, Mohakhali, Dhaka 1212, Bangladesh; saiful.sazed@icddrb.org (S.A.S.); golam.kibria@icddrb.org (M.G.K.); mshossain@icddrb.org (M.S.H.); fahad.zamil@icddrb.org (M.F.Z.); pranob.chandra@icddrb.org (P.C.A.); enayet.hossain@icddrb.org (M.E.H.); dahmed@icddrb.org (D.A.); rhaque@icddrb.org (R.H.)

**Keywords:** SARS-CoV-2, COVID-19, diagnostics, rapid test, PCR, surveillance, pandemic, rapid antigen detection, 2019 novel coronavirus

## Abstract

Accurate diagnosis at the right moment is the prerequisite for treatment of any disease. Failure to correctly diagnose a disease can result in highly detrimental effects, unmistakably a crucial factor during the COVID-19 pandemic. RT-PCR is the gold standard for COVID-19 detection while there are other test procedures available, such as LAMP, X-Ray, and ELISA. However, these tests are expensive, require sophisticated equipment and a highly trained workforce, and multiple hours or even days are often required to obtain the test results. A rapid and cheap detection system can thus render a solution to the screening system on a larger scale and be added as an aid to the current detection processes. Recently, some rapid antigen-based COVID-19 tests devices have been developed and commercialized. In this study, we evaluated the clinical performance of a new rapid detection device (*OnSite*^®^ COVID-19 Ag Rapid Test by CTK Biotech Inc., Poway, CA, USA) on COVID-19 symptomatic patients (*n* = 380). The overall sensitivity and specificity were 91.0% (95% CI: 84.8–95.3%) and 99.2% (95% CI: 97.1–99.9), against gold standard RT-PCR. The kit was capable of detecting patients even after 06 days of onset of symptoms and the sensitivity can be maximized to 98% in samples with an average RT-PCR Ct ≤ 26.48, demonstrating a high potential of the kit for clinical diagnosis of symptomatic patients in healthcare facilities.

## 1. Introduction

Thousands of people are losing their lives every day due to the global COVID-19 pandemic, and fast and accurate diagnosis is still a barrier in most countries [1]. While this pandemic is not under control, it has already caused over 232 million confirmed cases and more than 4.7 million deaths worldwide [2]. Lack of expertise, false-negative results, and a relative scarcity of convenient diagnostic methods, worsen the situation [3]. Moreover, asymptomatic individuals are roaming around the communities, acting as a potential virus reservoir and thus posing a significant threat to community-level transmission [4,5]. Multiple vaccines have been deployed worldwide and others are in development, but still not readily available to everyone, and especially with the constant appearance of new viral variants, there are chances of transmission even after getting vaccinated [6,7,8]. Although, until now, no confirmed medicine can effectively treat patients infected with the SARS-CoV-2 virus [9,10], safe distancing, proper hygiene and early supportive treatment during active infection may often help prevent worst-case scenarios for an individual, as well as reduce community transmission [11]. Additionally, to understand more about the etiology of the disease, better testing accuracy and repeatability are needed to effectively screen people and take the necessary measures. Aggressive screening will contribute to the containment of the virus as well as community transmission [12,13]. Optimum solutions for mass-level screening are therefore urgently needed.

Several methods have been invented around the globe, including the RT-PCR, loop mediated isothermal amplification (LAMP), enzyme-linked immunosorbent assay (ELISA), rapid diagnostic test (RDT), radiological evaluation, and some mobile applications [14,15,16,17,18]. Every one of these methods has its flaws in terms of techniques, skills, resources and cost-effectiveness. While RT-PCR has been used as a gold standard, none of the other methods are being employed to that extent due to a lack of data and reliability [19]. However, RT-PCR is usually not fast enough, quite expensive and needs proper technical expertise, and thus, is not suitable for mass-level surveillance [20].

An alternative approach would be loop mediated isothermal amplification (LAMP) that like PCR detects the viral nucleic acids, with the advantage of the capacity to test at the field level in remote areas [20]. However, major issues arise as the process relies primarily on indirect methods like turbidity and a slight color changes that can cause incorrect results and may vary from person to person in the interpretation of the result, both qualitatively and quantitatively [21]. X-rays and computed tomography (CT) are often used medically to determine the damage caused in the lungs but may not be confirmatory to COVID-19, as there are other possible causes of results resembling COVID-19, such as pneumonia, bronchitis, and other chronic obstructive pulmonary diseases (COPDs) [22]. Furthermore, the test itself suffers from expensive equipment and difficulty in the analysis of the result. Other methods such as ELISA could have been a good option, if not for the pre-requisite for a reader and skilled personnel, as well as less than ideal time to result.

All of these aforementioned issues can be avoided by using a rapid and cheap diagnostic method such as the immune-chromatographic test (ICT) in the form of RDT cassette, that can be used in the field level as well as individually, as the test is relatively easy to use, not requiring skilled laboratory professionals. In this study, we performed a prospective evaluation of a new antigen-based lateral flow, qualitative immunoassay device (*OnSite*^®^ COVID-19 Ag Rapid Test) for detection of COVID-19 in symptomatic suspected patients in urban population as well as in diagnostic center to evaluate its clinical performance and the prospects for use as a tool for early, rapid, cheap, and efficient diagnosis of COVID-19.

## 2. Materials and Methods

### 2.1. Patients

Consenting individuals with symptoms of COVID-19 reported to the International Center for Diarrhoeal Disease Research Bangladesh (icddr,b) laboratory services unit (LSU) in Dhaka, Bangladesh for a COVID-19 RT-PCR test between 29 April and 15 September of 2021 were considered for this study. Participants who met the study inclusion criteria (recent fever and two symptoms) were offered to participate in this study. 380 patients with evident clinical COVID-19 symptoms were enrolled in this study for testing by healthcare professionals. The study physician checked and recorded the health status of the participant in a pre-structured form. 

### 2.2. Sample Collection

Both nasopharyngeal and oropharyngeal samples were collected from each study participant and pooled by an expert medical technologist at the designated COVID-19 sample collection booth maintained by the LSU in a single 3 mL Viral transport medium (VTM) for RT-PCR testing. For testing on the *OnSite* COVID-19 Ag Rapid Test, one nasal swab was sample collected by inserting the swab up to 2 cm inside the nostril, following the product’s Instruction for Use (IFU).

### 2.3. Onsite^®^ Rapid Test

The OnSite^®^ COVID-19 Ag Rapid Test recognizes SARS-CoV-2 Nucleocapsid Protein. Using ELISA and peptide scanning, the epitopes recognized by the test have been mapped to the N-terminal domain (NTD) of the Nucleocapsid Protein. It is a lateral flow chromatographic immunoassay. The test cassette consists of: (1) a colored conjugate pad containing anti-SARS-CoV-2 antibodies conjugated with colloidal gold (antibody conjugates) and (2) a nitrocellulose membrane strip containing a test line (Ag line) and a control line (C line). The test line is pre-coated with anti-SARS-CoV-2 antibodies, and the C line is pre-coated with control antibodies. The OnSite^®^ COVID Ag rapid test was performed on the spot as per the manufacturer’s IFU. Roughly in this study, a nasal swab is inserted into the nostril until resistance is met at turbinates (less than an inch to nostril) with gentle rotation several times against the nasal wall. The process is repeated in the second nostril to ensure that sample is collected, and the swab is then removed and inserted into an extraction buffer. The extracted sample is then dropped into the sample well of the cassette to finish the test procedure. When applied to the sample well, the extracted specimen migrates across the test strip by capillary action. SARS-CoV-2 antigen, if present in the extract, binds to the antibody conjugates, and the immunocomplex is then captured on the membrane by the pre-coated anti-SARS-CoV-2 antibody, forming a colored Ag line that indicates a COVID-19 positive test result. For each patient, RDT was performed immediately after sample collection, and result reading was performed at 15 min after adding the extracted sample to the test cassette.

### 2.4. RT-PCR for COVID-19 Diagnosis

RT-PCR testing was carried out within 24 h of sample collection by the Molecular Diagnostic Laboratory of LSU, an ISO 15189 and ISO 15190 accredited facility, and results were submitted to the national database. The test was performed targeting SARS-CoV-2 N1 and N2 genes, with human RNaseP as assay control target, after standard RNA extraction by MagMAX™ Viral/Pathogen II (MVP II) nucleic acid isolation kit using King Fisher Flex high-throughput automated extraction system (Thermo Fisher Scientific, Waltham, MA, USA). Primers recommended by Center for Disease Control (CDC) were used for the RT-PCR assay. In brief, we used Thermofisher TaqPath™ 1-Step RT-qPCR mastermix (Thermo Fisher Scientific, Waltham, MA, USA), no ROX. following a developed multiplex RT-PCR program on an ABI 7500 Fast DX instrument (Thermo Fisher Scientific, Waltham, MA, USA). 20 μL mastermix containing 5 μL of template was used for each test. The reaction condition includes steps of cDNA synthesis (10 min/55 °C), a hold step (1 min/95 °C), and subsequently 45 cycles of denaturation (10 s/95 °C) and annealing/elongation (30 s/55 °C) [23]. The threshold for a positive outcome was considered 40 amplification cycles.

### 2.5. Sanger Sequencing and Clade Identification

For SARS-CoV-2 circulating variant screening, we sequenced spike protein using specific primer sets from ARTIC nCoV-2019 V3 panel. Among the 133 RT-PCR positive samples, 69 samples with low Ct (<27.00) values were sequenced. Sanger sequencing was performed in an automated ABI 3500 XL genetic analyzer (Applied Biosystems, Foster City, CA, USA) described elsewhere [24]. The raw sequence data were inspected by BLAST search; contigs were mapped using the SeqMan tool (DNASTAR, Inc., Software, Madison, WI, USA). The SARS-CoV-2 clade was detected using the Nextclade v1.5.2 (https://clades.nextstrain.org/ (accessed on 3 October 2021)) with default parameters [25].

### 2.6. Ethical Considerations

The study was approved by the institutional ethical committee (ERC) of the International Center for Diarrhoeal Disease Research, Bangladesh (icddr,b). At the time of enrollment, written informed consent was obtained from each adult participant. For the children aged 2–10 years, consent was obtained from the parents or legal guardian and in the cases of children between 11–17 years in addition to written informed consent, verbal assent was also obtained.

### 2.7. Data Analysis

Data were computed in an Excel sheet. Statistical analysis was carried out using GraphPad Prism 8.0.2 (GraphPad Software, Inc., San Diego, CA, USA) and Stata version 15.1 (StataCorp, College Station, TX, USA) were used for all analysis. Paired Student’s t-test was used to compare the mean and Wilcoxon rank-sum (Mann-Whitney) test was used to compare the non-normally distributed continuous data. To calculate sensitivity, specificity, positive predictive value (PPV) and negative predictive value (NPV) and respective confidence intervals, diagti command was used. Receiver operating characteristics (ROC) curves were built using MedCalc Statistical Software version 20.013 (MedCalc Software, Ostend, Belgium) for the analysis of sensitivity and the specificity of the RDT. A heat map was also generated to evaluate the relation among symptoms, days passed after the onset of the symptoms as well as the ability of the rapid test to detect COVID-19 at various time points.

## 3. Results

### 3.1. Enrollment

A total of 380 individuals were enrolled where 230 (60.5%) were male with the median (IQR) age 33 (27–42) and 150 (39.5%) were female with the median age 32.5 (25–42). There was no significant difference between median age (*p* = 0.26).

### 3.2. COVID-19 Patients Confirmed by RT-PCR

Out of 380 patients, 133 (35.0%) tested positives in RT-PCR where Ct values for N1 and N2 ranged from 11.0–31.7 (mean ± sd: 18.2 ± 4.7) and 11.0–36.1 (mean ± sd: 19.1 ± 5.0) respectively. The mean difference between the Ct values for N1 and N2 was significant (*p* < 0.001)

### 3.3. Performance of the Onsite COVID Ag RDT

**Figure 1:**
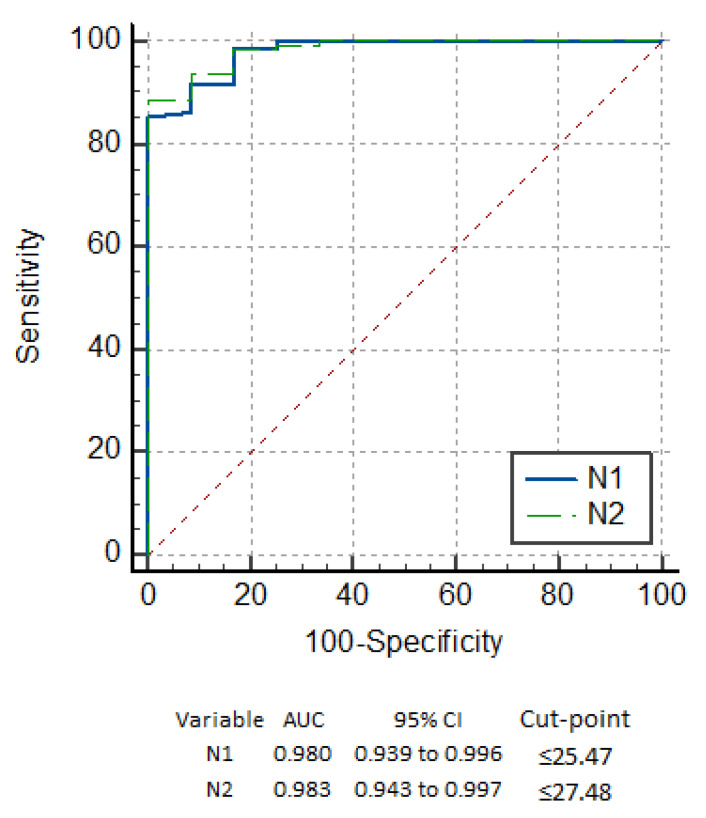
ROC curve for COVID-19 Ag RDT at cut-point where maximum sensitivity and specificity achieved for N1 and N2 targets.

Among the 133 study participants classified as positive by RT-PCR, 121 (91.0%) tested positive by the *OnSite*^®^ COVID-19 Ag RDT. Among the 247 study participants classified as negative by RT-PCR, 245 (99.2%) tested negative by the *Onsite*^®^ COVID-19 Ag RDT. The kit’s sensitivity and specificity values in this study were therefore 91.0% (95% CI: 84.8–95.3%) and 99.2% (95% CI: 97.1–99.9) respectively. The positive and negative predictive values (PPV and NPV) are 98.4% (95% CI: 94.2–99.8%) and 95.3% (95% CI: 92.0–97.6%). When comparing the performance of the *OnSite*^®^ COVID-19 Ag RDT with RT-PCR, it shows a very high concordance (agreement = 96.3, κ = 0.92, *p* < 0.001), as depicted in the ROC curve in Figure 1.

**Figure 2:**
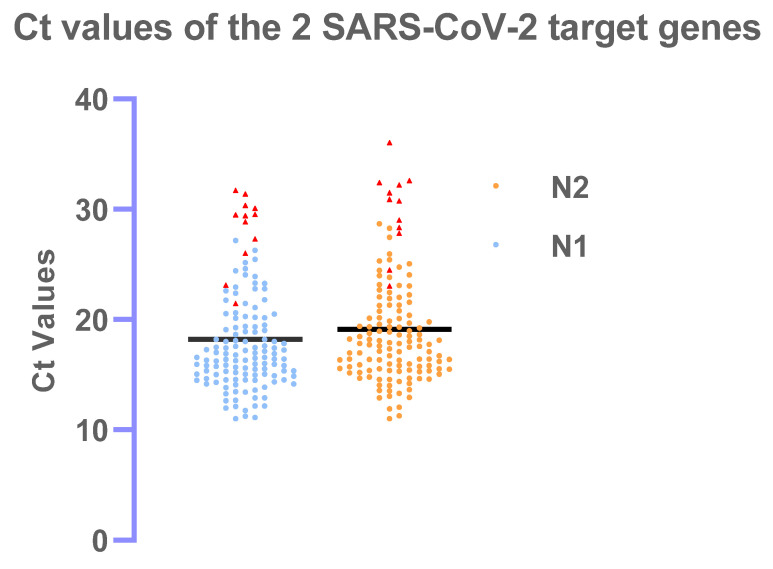
Scatter diagram showing distribution of RT-PCR positive patients for the SARS-CoV-2 N1 and N2 targets with individual Ct values. The red triangles represent the RT-PCR positive samples that have been missed by the Onsite^®^ COVID-19 Ag RDT.

The RDT detected all but 2 positive samples with Ct values below 25.15 for the N1 and 25.93 for the N2 target. The other samples where the RDT failed to detect a positive result, had Ct values >26 and, in the majority of the cases >29.0, corresponding to samples with low viral load, and therefore with a lower infectious potential. These cases have been picturized in the Figure 2 as a scatter diagram with red triangles indicating positive samples that the RDT missed. In addition, the true positive and false positive rates have been summarized in the ROC curve in Figure 1. The maximum performance of the kits Onsite^®^ COVID-19 Ag RDT found at the cut-point 25.47 for N1 and 27.48 for N2 with sensitivity of 98.35% (Figure 1).

**Figure 3:**
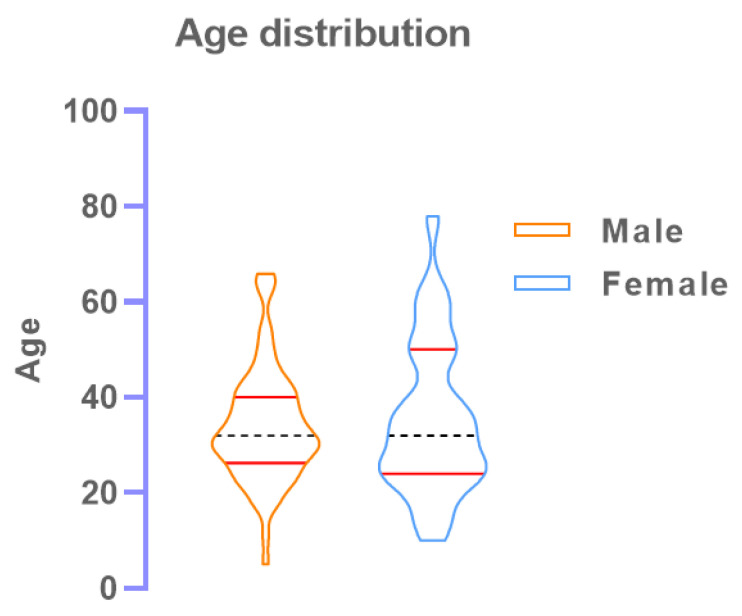
Violin curve depicting the various age group of participants who were RT-PCR (+ve). The red scattered circle points represent the RT-PCR positive samples only.

A distribution of the RT-PCR positive patients’ age in the study is shown as a violin curve (Figure 3). The graph indicates all age groups of patients getting infected by SARS-CoV-2.

### 3.4. Detection Based on Symptoms

**Figure 4:**
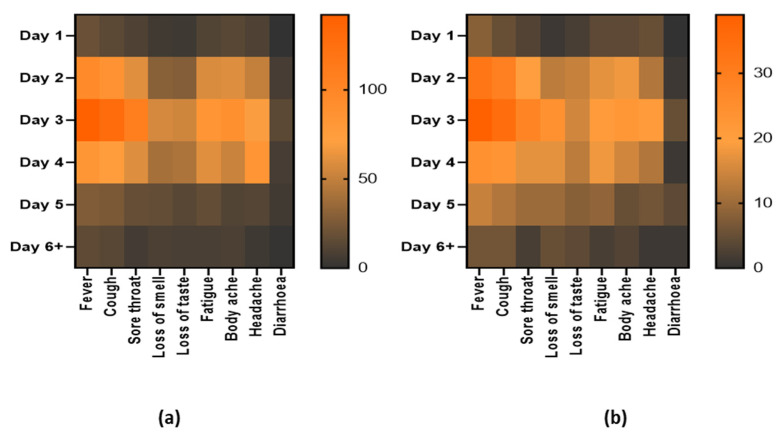
Heat maps representing the major symptoms of patients. From day 1 to day 6 and above post onset of symptoms for (**a**) patients coming to the diagnostic facility for the test and (**b**) patients who are positive by the *OnSite*^®^ COVID-19 Ag RDT. The right-sided bar in each heat map represents the number of patients in each group.

Overall, a symptoms-based analysis has also been displayed through the heat map in Figure 4. Nine (9) major COVID-19 symptoms were taken into consideration with the days passed post-onset of symptoms and the number of patients in the bar associated with each heat map (Figure 4a). Besides, the same correlation has been depicted in the heat map of Figure 4b for only the positive patients by RDT. The primary symptoms are fever, cough, sore throat, loss of smell, loss of taste, fatigue, body ache, headache, and diarrhea. The frequency of overall patient arrival and positive samples have been distributed among day 1 to day 6 and above.

### 3.5. Detection Based on Variants

**Figure 5:**
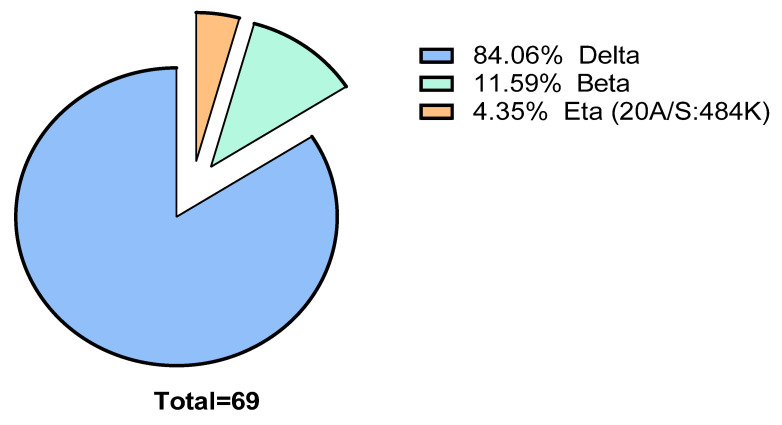
A pie chart depicting the circulating variants during the study. It also represents the variants among the RT-PCR and RDT positive patients who came to the diagnostics facility in the particular study time frame.

Among the 69 samples, 58 were delta (Indian) variant (84.06%), 8 beta (South African) (11.59%) variant and 3 eta (20A/S:484K probably Nigerian) variant (4.35%). The RDT could detect all the samples that were sequenced indicating the capability to detect different variants of SARS-CoV-2. The summary has been depicted in the Figure 5.

## 4. Discussion

Several rapid test devices have been evaluated so far in various platforms, and most of those were conducted either with archived samples, samples preserved in VTM or a limited sample size for prospective studies [26,27,28,29,30]. In this study, we evaluated the practicality of implementing the use of the *OnSite* COVID-19 Ag rapid test kit as an easy-to-use test to detect COVID-19. The high sensitivity and specificity of the *OnSite* COVID-19 Ag rapid test obtained in this study, indicate a very robust performance of the test kit. The RT-PCR positive samples that have been missed by RDT, have mostly very high Ct values (>27) (Figure 2). Only two samples with RT-PCR results below Ct value of 25 have been missed, which might have been a result of improper sample collection, dried nostrils or others. Supporting this hypothesis, the 12 samples that produced false negative results versus the RT-PCR assay, stored in VTM after collection for the RT-PCR assay, were later re-tested on the *OnSite* COVID-19 Ag rapid test, and 3 out of these 12 samples produced positive results, including the 2 RT-PCR positive samples of Ct < 25.0 in the test cassette. This could also indicate that using nasopharyngeal swab samples might result increased sensitivity compared to the nasal swab, as there is a greater chance of discharged secretion of the specimen from nasopharyngeal mucosa. As all the tests are dependent on proper sample collection, the false-negative results might have been explained by the improper discharge from respiratory mucosa or by low viral load. Only two false positive results were obtained in 380 samples, substantiating the high specificity of the *OnSite* COVID-19 Ag rapid test kit 99.2% (95% CI: 97.1–99.9). The false positive samples were tested with an additional RT-PCR protocol and reconfirmed as a true negative. These samples were also negative for other COVID RT-PCR target i.e., SARS-CoV-2 virus genome, E-gene, N-gene and RdRp gene [31,32]. 

A study evaluating the performance of Panbio^TM^ COVID-19 Ag Rapid Test Device (Abbott Rapid Diagnostics, Lake Country, IL, USA) and Standard^TM^ Q COVID-19 Ag Test (SD Biosensor, Osong, Korea) found the overall sensitivity of these kits were 89% and 85.5%, respectively, and with specificities of 99.7% and 100% respectively [33]. Another study that evaluated the Panbio^TM^ COVID-19 Ag Rapid Test Device found the sensitivity and specificity of 79.6% and 100%, respectively. It was found that RDT negative samples were not infectious although positive by RT-PCR. However, the number of the positives samples in this study was small (only 43 samples positive by both RT-PCR and rapid test with 11 discordant results out of 412) [34]. The Panbio^TM^ COVID-19 Ag Rapid Test Device was also used in a prospective study with 634 asymptomatic patients in Spain, of whom 79 were positive, and the kit was able to detect in 48.1% cases only (38 patients) [35]. However, Standard™ Q COVID-19 Ag kit (SD Biosensor^®^, Osong, Republic of Korea) was compared with RT-PCR of Allplex™ 2019-nCoV Assay (Seegene^®^, Seoul, Korea) among 454 patients of which 60 people were positive and found 98.33% sensitivity. This high sensitivity might be explained by the small sample size in the study, and mainly by the low sensitivity (90%) of the RT-PCR kit, which will likely miss the low viral load samples, that would also be missed by the rapid test [36]. However, in the WHO evaluation of the SD Biosensor kit, the observed pooled sensitivity was 84.97% (130/153, 95% CI 78.3–90.23%) and the pooled specificity was 98.94% (1490/1506, 95% CI 98.28–99.39%) [37].

From the ROC curve it is evident that maximum sensitivity of 98.35% can be obtained with the *OnSite* COVID-19 Ag rapid test kit in case of Ct values of 25.47 and 27.48 for N1 and N2 target respectively. Comparatively, AFIAS COVID-19 Ag test and ichroma^TM^ COVID-19 Ag test (Boditech Med., Chuncheon-si, Gang-won-do, Republic of Korea) showed 100% sensitivity and specificity for Ct values <25.0 for RdRp and N gene. However, for E gene, the sensitivity falls to 91.3% and 95.7% respectively. For Ct values ranging from 25–30, the sensitivity reduces drastically to 34–64.4% [38]. In another study, overall 79% sensitivity was obtained with area under the curve (AUC) of 0.88 with an optimal Ct cut-off value of 29 whereas in this study an AUC of 0.98 for an average cut-off value of 26.48 was obtained.

This *OnSite* COVID-19 Ag Rapid Test kit showed high performance in determining positive patients that came to test after 1 to >6 days from the onset of symptoms. The heat map also indicates that patients of all age groups had come to the test facility as well as that the positive samples are well distributed among the age ranges. However, fever, cough, sore throat, and body ache seemed prominent symptoms among the COVD-19 positive patients. The result also portrays its capability to detect major circulating variants of concern (VOC) as well as variants being monitored (VBM).

In the community and healthcare facilities, it often takes a significant amount of time from sample collection to RT-PCR test, from multiple hours to often even days. There is also a chance of cross-contamination of the samples that may render false-positive results when testing in batch form. One of the common pitfalls of RT-PCR is that it is also costly, and require skilled personnel along with sophisticated equipment, making it unavailable in resource-limited settings. In contrast, the *OnSite* COVID-19 Ag Rapid Test is a cost-effective easy to use test that does not require specialized equipment or skilled laboratory personnel. Besides, there is minimal chance of cross-contamination of samples, as the kit is designed for individual patients and limits hazardous features to the operator, as the virus is inactivated by the extraction buffer media. The high sensitivity and specificity of the kit also justifies its ability to be a viable alternative to RT-PCR.

The main limitation of the rapid tests is that there is possibility of false negative results in samples with low viral load. Hence, it might not be often suitable for detection of COVID-19 in less symptomatic and asymptomatic patients. 

## 5. Conclusions

Many countries worldwide are now shifting towards rapid Ag-based detection systems in the healthcare facilities for diagnosis of COVID-19, to reduce time as well as costs. However, RDT negative symptomatic samples should be confirmed by RT-PCR. It is likely that nasopharyngeal samples have slightly better sensitivity (92.48%) than nasal swabs only, but sample collection is less invasive to the patient when using nasal swabs. The sensitivity obtained for nasal samples should be comparable to self-testing performance at the community level, whereas nasopharyngeal sample could be an alternative for professional testing at the healthcare facilities with improved diagnostic capacity. Our study demonstrates that the *OnSite* COVID-19 Ag Rapid Test is a very reliable, fast, user-friendly and economic test, that can easily be implemented as the test of choice for rapid detection of COVID-19 in all sorts of healthcare facilities.
